# The effect of luck perception on intertemporal decision-making: the mediating role of decision confidence and the moderating role of self-construal

**DOI:** 10.3389/fpsyg.2025.1620033

**Published:** 2025-11-04

**Authors:** Hao Zhong, Yuwei Wang, Yanxia Li

**Affiliations:** ^1^Research Center for Ecological Civilization, School of Economics and Management, Gandong University, Fuzhou, Jiangxi, China; ^2^School of Education, Nanchang Institute of Science and Technology, Nanchang, Jiangxi, China

**Keywords:** luck perception, intertemporal decision-making, decision-making confidence, self-construal, delayed reward

## Abstract

Lucky cues represent a common yet subtle situational stimulus in daily life. They can instill confidence and optimism in individuals, enabling them to seize opportunities and address challenges effectively. Moreover, lucky cues significantly influence consumption behaviors, diversity seeking, novelty pursuit, and risk-taking tendencies. However, prior studies have rarely examined luck within uncertain contexts or integrated its unique positive psychological resources to explore its effects on intertemporal decision-making. To address this gap, we conducted three experimental studies to investigate the relationship between individual luck perception and intertemporal decision-making from an uncertain intertemporal perspective. We revealed that perceiving luck would enhance individuals’ tendency to choose delayed gratification in intertemporal decision-making tasks. Furthermore, decision-making confidence would mediate this effect. Specifically, compared with the bad-luck group, individuals in the good-luck condition would exhibit stronger decision confidence and demonstrate a greater preference for delayed option in intertemporal decision-making. Additionally, self-construal would moderate both the direct impact of luck perception on intertemporal decision-making and the mediating role of decision confidence. For individuals with an interdependent self-construal, luck perception would strengthen their decision confidence, making them more likely to select larger-later rewards in intertemporal decision-making. In contrast, this effect would be weaker among individuals with an independent self-construal. This study extend the understanding of luck perception in intertemporal contexts and provide practical implications for interventions in education, decision-making guidance, and psychological correction.

## 1 Introduction

Intertemporal decision-making refers to the cognitive process through which individuals evaluate and trade off costs and benefits occurring at different points in time (Guo et al., 2024). This fundamental decision-making mechanism operates across multiple domains, ranging from everyday personal choices—such as selecting between fried kebabs and vegetable salads or deciding whether to stay up late using mobile devices versus adhering to a regular sleep schedule—to broader organizational and societal decisions, including technology acquisition versus independent innovation and resource-intensive versus environmentally sustainable development strategies (Fagerholm et al., 2023; Colton et al., 2024; Qu et al., 2024). As such, individuals routinely face the central dilemma of whether to prioritize immediate gratification or long-term outcomes. A substantial body of research has identified various determinants of intertemporal choice. These include individual differences such as future self-continuity and mortality awareness (Hershfield, 2011; Yamamori et al., 2018; Wang and Wang, 2024), attributes of choice options such as temporal delay and outcome magnitude (Chen and He, 2014; Pei et al., 2019; Yamamori et al., 2018; Yin et al., 2022), and situational influences such as monetary cues and inspirational stimuli (Guo et al., 2024; Keidel et al., 2021). However, one pervasive yet underexplored contextual factor is the luck perception. The role and underlying mechanisms of luck in shaping intertemporal preferences remain poorly understood.

Existing research has predominantly examined luck within risky or uncertain contexts (e.g., Jiang et al., 2009), demonstrating that luck perception increases risk-seeking behavior. However, this focus implicitly conflates “luck” with “risk,” thereby overlooking a critical question: Does luck perception influence decision-making even when outcomes are certain—as in standard intertemporal choices where delayed rewards are guaranteed? Furthermore, does its psychological mechanism differ from that observed in risky decision-making?

Addressing this gap thus extends the conceptual scope of luck research from the probabilistic to the temporal domain, thereby enhancing our understanding of why seemingly irrelevant cues, such as lucky draws or symbolic mascots, systematically shape future-oriented behaviors (e.g., saving, educational investment) in daily life, which is largely characterized by non-probabilistic decisions.

Moreover, recent research indicates that, consistent with Construal Level Theory, luck perception activates abstract cognitive processing, leading individuals to focus more on global meaning and long-term value (Tao et al., 2023), which may increase their preference for delayed rewards. In contrast, alternative perspectives suggest that the “pleasant surprise” associated with luck perception could amplify hedonic motivation, thereby encouraging present-oriented consumption and impulsive enjoyment of immediate fortune (Jiang et al., 2021). To further clarify the role of luck perception in intertemporal decision-making, this study integrates Construal Level Theory and Supernatural Agent Theory, drawing on the Broaden-and-Build Theory as a foundational perspective to develop a more comprehensive theoretical framework. We propose that the effect of luck perception on intertemporal choices is mediated by decision-making confidence—a central yet unexamined psychological mechanism.

Specifically, we posit the following causal chain: Luck perception, as a positive self-relevant experience, can substantially strengthen individuals’ positive self-perceptions and sense of efficacy (Darke and Freedman, 1997). This favorable self-assessment strengthens individuals’ confidence in their ability to make sound judgments—i.e., decision confidence (Jiang et al., 2009; Tao et al., 2023). In intertemporal contexts, choosing a delayed reward requires tolerating short-term costs while maintaining belief in the eventual realization of larger future gains. Decision confidence acts as a psychological stabilizer in this process: individuals with higher decision confidence exhibit greater tolerance for temporal uncertainty (e.g., “Will I actually receive the future reward?”) and stronger conviction in the attainability of future benefits. Consequently, they are more likely to adopt patient, future-oriented choices. We therefore hypothesize that decision-making confidence mediates the relationship between luck perception and increased preference for delayed rewards.

Importantly, this mediating process may vary across individuals depending on their chronic self-construal. According to self-construal theory, individuals with an independent self-construal, who emphasize autonomy and internal attributes, typically base their decision confidence on stable, intrapersonal beliefs. As such, they are less likely to rely on the perception of luck as a psychological resource to bolster self-assurance. In contrast, individuals with an interdependent self-construal, whose sense of security and self-confidence is more contingent on external social connections, may lack robust internal reference points when making decisions. Consequently, they are more prone to seek support from external cues—such as the luck perception—thereby exhibiting a stronger psychological dependence on such factors (Markus and Kitayama, 1991). Therefore, we hypothesize that the effect of luck perception on intertemporal decision-making will be more pronounced among individuals with an interdependent self-construal.

In conclusion, this study aims to systematically elucidate the underlying mechanisms and boundary conditions through which luck perception influences intertemporal decision-making, by employing a moderated mediation framework. Theoretically, this research extends the domain of luck-related inquiry from risk-laden contexts to intertemporal choices with deterministic outcomes. Furthermore, it represents the first attempt to integrate the theoretical perspectives of Construal Level Theory and Psychological Force Theory. By positioning decision-making confidence as the central mediating mechanism and self-construal as a key moderating factor, the present work advances a novel integrative model capable of reconciling divergent findings in the extant literature. Practically, the results offer meaningful and forward-looking implications. They can inform the design of educational interventions intended to foster long-term orientation, support the development of personalized communication strategies based on individual differences in self-construal within financial and consumer settings, and contribute to the refinement of confidence-building therapeutic approaches for addressing addictive or impulsive behaviors.

## 2 Theoretical background and research hypothesis

### 2.1 Luck perception and intertemporal decision making

Luck in life generally refers to good fortune or being fortunate, which occurs when the outcome of an event or behavior aligns with an individual’s expectations. In early psychological research grounded in attribution theory, luck was considered an elusive and unpredictable random factor that exerted minimal influence on outcomes. Compared to internal factors such as ability and effort, this sporadic random factor was deemed to have a relatively minor impact on personal development (Zhao and Zhong, 2022). However, in real-world contexts, some individuals do not perceive luck as a purely random probabilistic factor. Based on this observation, scholars in the personality trait school proposed the trait luck perspective, suggesting that luck is a relatively stable personal characteristic that can provide positive psychological resources such as optimism, confidence, and hope. For example, individuals who believe in luck often expect it to favor them and hold strong positive expectations regarding outcomes (Darke and Freedman, 1997; Wang et al., 2021). Recent social psychology research has demonstrated that the concept priming effect plays a crucial role in influencing individual cognition and behavior. Similar to memory, people’s understanding of concepts is stored in the brain for extended periods and can be activated in specific situations, thereby affecting their psychology and behavior in unrelated contexts (Sun et al., 2024). Given the long-term availability of luck, the concept of luck—comprising experiences, feelings, beliefs, or items—can be retained in deep memory over time. Through cognitive priming processes, this retention stimulates an awareness of luck, generating a temporary luck perception (Jiang et al., 2009). Regardless of whether an individual believes in luck, when the concept of luck is activated, they are unconsciously influenced by luck-related cues during decision-making, which in turn affects their subsequent behavioral choices (Wang et al., 2021; Jiang et al., 2021). In summary, luck represents a prevalent cultural phenomenon in people’s daily lives. Moreover, as an integral part of an individual’s stable traits, luck can endow individuals with confidence and optimism, exerting a substantial influence on their consumption choices, behavioral decision—making, and other aspects. Luck perception, on the other hand, refers to the subjective perception that an individual experiences upon being stimulated by luck—related cues. It is one of the irrational beliefs associated with luck and serves to depict the perception of a stable external force. This perception has the ability to motivate individuals to form positive anticipations regarding future events and instill in them the belief that they can achieve success within uncertain circumstances.

Intertemporal decision-making entails evaluating outcomes distributed across time, a process that inherently involves trading off immediate against delayed rewards (Frederick et al., 2002). Whereas its research tradition has focused on temporal discounting (i.e., the devaluation of future outcomes due to delay; Mischel et al., 1989), risk decision-making concerns choices under probabilistic uncertainty (Huang et al., 2023). Nevertheless, despite being defined by time versus probability, converging evidence demonstrates notable overlaps in their underlying psychological representations and neural substrates (Luckman et al., 2020; Hardisty and Pfeffer, 2017). A key reason for this convergence lies in the inherent uncertainty embedded in delayed rewards: the passage of time introduces doubts about whether the reward will be delivered as expected or retain its value (“Will I receive it?” or “Will it lose worth?”). As a result, individuals often apply a discounting mechanism similar to that used for probabilistic outcomes, with both time and probability contributing to the broader construct of “psychological distance,” thereby amplifying perceived uncertainty (Sun and Li, 2010; Chen and He, 2012).

This raises a critical question: How does luck perception shape decision-making under such temporally induced uncertainty? The existing literature presents an apparent contradiction. On one hand, empirical findings suggest that heightened luck perception increases risk-seeking tendencies—for instance, leading individuals to purchase new products immediately rather than wait for price reductions (Zhao et al., 2016). Yet this effect is predominantly observed in contexts involving probabilistic risks, where outcomes are inherently uncertain. In contrast, the present study focuses on intertemporal decisions with certain outcomes, where the primary source of uncertainty stems not from outcome probability but from the psychological strain of waiting. Drawing on Construal Level Theory, we propose that luck perception activates high-level, abstract construal’s, directing attention toward overarching meanings and long-term goals (Tao et al., 2023). Concurrently, the Supernatural Agent Theory suggests that perceiving oneself as lucky enhances self-concept clarity and fosters optimistic expectations about future outcomes (Lim and Rogers, 2017). Supporting this, research shows that situational luck induction boosts optimism and confidence in uncertain tasks, encouraging exploratory behavior, risk-taking, and enhanced creativity (Zhao, 2023). Individuals experiencing high luck perception exhibit greater willingness to purchase lottery tickets and allocate larger investments in risky financial decisions. In consumer contexts, they show a stronger preference for low-probability, high-value promotional lotteries over guaranteed small-value coupons (Jiang et al., 2009; Ke et al., 2024). Therefore, we argue that in intertemporal decision-making, luck perception mitigates the psychological burden associated with waiting by providing both a long-term cognitive orientation and enhanced confidence in future outcomes. As a result, individuals are more likely to forgo immediate rewards and opt for larger, delayed, yet certain benefits.

*H1*: Luck perception influences individuals’ intertemporal decision making. Specifically, individuals who perceived good luck, as compared to those who perceived bad luck, are more likely to prefer delayed rewards in intertemporal choices.

### 2.2 The mediating role of decision-making confidence

In intertemporal decision-making, delayed options with greater value are often associated with higher uncertainty or risk due to the extended time horizon. Consequently, individuals facing intertemporal choices need sufficient decision-making confidence to reduce their perception of uncertainty and facilitate optimal decision-making (Golman et al., 2021; Xue et al., 2024). Decision-making confidence refers to an individual’s subjective certainty regarding the correctness and optimality of their decisions (Peterson and Pitz, 1988), serving as a critical mediator between decision intention and behavioral enactment (Tsai and McGill, 2011). In intertemporal decision-making, choosing a delayed reward requires not only the ability to forgo immediate gratification but also confidence in one’s capacity to endure the waiting period and trust in the eventual realization of greater future value. Thus, decision-making confidence constitutes a fundamental psychological mechanism that supports patient, future-oriented choices (Golman et al., 2021).

We propose that decision-making confidence is the central mediating pathway through which luck perception influences intertemporal decision-making. First, according to the Broaden-and-Build Theory (Fredrickson, 2001), perceiving oneself as lucky serves as a positive emotional experience that builds enduring personal resources such as self-confidence and optimism (Au et al., 2011; Hock et al., 2020). Thereby functioning as a positive self-relevant cue, luck perception enhances an individual’s self-efficacy and sense of control over future outcomes, which in turn strengthens decision-making confidence (Darke and Freedman, 1997; Zhao, 2023). Second, drawing on the Supernatural Agent Theory, feeling lucky evokes a psychological sense of being supported by an external, benevolent force (Damisch et al., 2010). This perceived “protection” reduces subjective uncertainty and reinforces a sense of agency, providing a stable cognitive-emotional foundation that bolsters confidence in one’s decisions (Hamerman and Morewedge, 2015).

Elevated decision confidence, in turn, directly facilitates preference for delayed rewards. Empirical evidence shows that individuals with higher decision confidence are better able to resist immediate temptations and exhibit stronger preferences for long-term beneficial outcomes (Kim et al., 2019; Sun et al., 2024). This occurs because increased confidence attenuates the perceived risk associated with delay and strengthens belief in the attainability of future rewards. While luck perception may concurrently elevate other affective states—such as general positive affect—decision-making confidence is uniquely positioned as a cognition-focused construct that directly reflects an individual’s belief in the validity of their choice and the likelihood of future success. As such, it aligns more precisely with the core requirement of intertemporal decision-making: the willingness to act on the belief that a better outcome will materialize in the future.

*H2*: Decision-making confidence mediates the influence of luck perception on intertemporal decision-making.

### 2.3 The moderating role of self-construal

Self-construal refers to a systematic cognitive framework through which individuals define their identities and establish relationships with others. It primarily comprises two orientations: independent self-construal, which emphasizes personal uniqueness, and interdependent self-construal, which highlights relational connectedness (Markus and Kitayama, 1991; Torelli, 2006). This self-concept profoundly shapes cognitive processing. Specifically, individuals with an independent self-construal tend to employ analytical thinking and rely predominantly on internal cues in decision-making, whereas those with an interdependent self-construal are more likely to adopt holistic thinking and exhibit greater sensitivity to environmental cues (Li et al., 2023; Hsieh et al., 2021; Shang et al., 2022).

This cognitive distinction implies that individuals with an interdependent self-construal are more susceptible to contextual influences such as luck. First, according to Construal Level Theory, the concept of luck influences consumers’ construal levels. Perceiving luck induces a higher-level construal, leading individuals to adopt a goal-directed mindset and prioritize abstract, overarching features and expected outcomes of behaviors or events. This shift in cognitive representation aligns closely with the holistic thinking pattern characteristic of individuals with an interdependent self-construal (Tao et al., 2023). Second, drawing on the supernatural agent theory, luck functions as an external mechanism that helps individuals mitigate environmental uncertainty and enhances their psychological resilience and decision-making confidence (Damisch et al., 2010). Critically, the attribution of luck differs fundamentally between self-construal types. For individuals with an interdependent self-construal, luck is not viewed as an isolated personal attribute but rather as a socially embedded opportunity tied to interpersonal networks and communal support. Consequently, feelings of luck are interpreted as signals of being situated within a favorable and supportive social environment. This perceived endorsement from external forces can significantly strengthen future-oriented decision-making confidence (Hamerman and Morewedge, 2015). In contrast, individuals with an independent self-construal are more inclined to internalize luck as a transient and unstable personal state. Because this attribution lacks stability and durability, it fails to serve as a reliable basis for sustained confidence in decision-making. As a result, the confidence-enhancing effect of luck perception is diminished, thereby attenuating its influence on intertemporal decision making.

*H3*: The influence of luck perception on intertemporal decision-making is significant among individuals with an interdependent self-construal, but is weakened among those with an independent self-construal.

*H4*: For individuals with an interdependent self-construal, luck perception strengthens decision-making confidence, thereby increasing the tendency to choose delayed options, while this effect is significantly attenuated among those with an independent self-construal.

**Figure 1:**
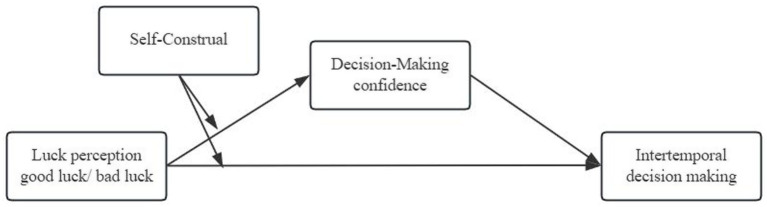
Conceptual research model.

Our conceptual research model is shown in Figure 1.

## 3 Experiment 1: luck perception and intertemporal decision making

### 3.1 Purpose and design of the experiment

The purpose of experiment 1 was to validate the effect of luck perception on intertemporal decision-making preferences (H1). In the experiment 1, A one-factor (luck prime: good luck vs. bad luck vs. control) experimental design was used. To ensure the validity of the sample size, the sample size was calculated to be at least 180 (*f* = 0.25, *α* = 0.05, 1 − *β* = 0.80) by G Power 3.1 software. All the participants of this experiment were college students. The recruitment information was mainly released through the online learning platform *Xuexitong* (the most commonly used learning platform for college students in Chinese mainland) or the *Confession Wall* (an important channel for college students in Chinese mainland to obtain and share information) on campus. The recruitment period for this study was from December 1, 2024, to December 7, 2024. To enhance the effectiveness of decision-making tasks, participants in experiment 1 need to have experience in using *Weibo* and participating in lotteries. Meanwhile, the subjective value calculation method of the delayed option in experiment 1 would exclude invalid decision-making choices, such as choosing data that repeats back and forth. A total of 310 college students from Gandong University in central China were actually recruited, among whom 20 were excluded because they made invalid choices in the decision-making task, *M*_age_ = 21.20, *SD* = 0.96, males: 127 (43.7%). Research involving human participants was reviewed and approved by School of Economics and Management, Gandong University. Participants provided written informed consent to participate in this study.

### 3.2 Experimental materials and methods

First, the participants were randomly allocated to three groups: the good luck condition (*n* = 76), the bad luck condition (*n* = 74), and two corresponding control groups (*n* = 140). The specific scenario is as follows: recently, a technology company is about to launch an ultra-thin fast-charging wireless magnetic power bank, in order to better determine the retail price, the company would like to find out what people’s psychological expectation of the price of this new rechargeable battery is. Meanwhile, to assist the public in their deliberation, the company would generate a random reference price for evaluation purposes. The reference price presented to the good luck condition was “88 RMB” [8 is the same as the pronunciation of “fa” (represents prosperity), which implies good luck]; while that for the bad luck condition was “44 RMB” (The number 4 is unlucky because it is pronounced like death in Chinese). The control condition was assigned neutral numbers. In order to eliminate the potential anchoring effect of these prices, two additional control groups were established for both the good-luck and bad-luck conditions. The average price values across these control groups were aligned with the respective experimental group prices. Specifically, the control condition corresponding to the good-luck condition randomly displayed “101 RMB” and “75 RMB,” whereas the control condition for the bad-luck condition randomly showed “57 RMB” and “31 RMB.” Participants were asked to indicate whether their psychological expected price for the product was higher or lower than the presented price. Additionally, a manipulation check was conducted to assess participants’ luck perception. Through the following statements: “I feel I have good luck,” “I feel today is my lucky day,” and “I feel I am lucky at this moment” (Cronbach’s *α* = 0.93).

Subsequently, participants were instructed to complete an intertemporal decision-making task under the following scenario. Drawing on the methodologies of Rachlin and Jones (2008) and Xu et al. (2019), participants were presented with the following situation: “You recently participated in a lottery event organized by the official Weibo platform. Today, you received a call from the *Weibo* staff informing you that you have won. You are now given the option to either receive a smaller reward immediately or wait 1 month to receive a larger reward. Please select your preference based on the options provided.”

This experiment presents nine sets of options, each consisting of two alternatives: an immediate reward or a delayed reward. The delayed reward in all sets is a fixed amount of 100 RMB to be received 1 month later. In contrast, the immediate rewards range from 10 RMB to 90 RMB, increasing incrementally by 10 RMB per set. Participants are required to make a choice between A (immediate reward) and B (delayed reward) for each of the nine sets.

**Table:** 

Group 1	A. Get 10 RMB now	B. Get 100 RMB after 1 month
Group 2	A. Get 20 RMB now	B. Get 100 RMB after 1 month
Group 3	A. Get 30 RMB now	B. Get 100 RMB after 1 month
Group 4	A. Get 40 RMB now	B. Get 100 RMB after 1 month
Group 5	A. Get 50 RMB now	B. Get 100 RMB after 1 month
Group 6	A. Get 60 RMB now	B. Get 100 RMB after 1 month
Group 7	A. Get 70 RMB now	B. Get 100 RMB after 1 month
Group 8	A. Get 80 RMB now	B. Get 100 RMB after 1 month
Group 9	A. Get 90 RMB now	B. Get 100 RMB after 1 month

The preference for delayed rewards is quantified by the subjective value assigned to the delayed option. The subjective value of the delayed option as an approximation of the present value of the delayed option, which the participant used to compare with the value of the immediate option. The subjective value of the delayed option was assessed using the procedure detailed in Rachlin and Jones (2008), which provides a validated framework for quantifying intertemporal preferences. Specifically, it was as follows: If a participant consistently selects A (or B) across all option groups, the subjective value of the delayed option is determined to be 5 RMB (or 95 RMB). If the participant initially selects B in earlier groups and subsequently switches to A in later groups, the subjective value of the delayed option is calculated as [(the amount of the immediate reward when A was first selected) + (the amount of the immediate reward in the preceding group)] ÷ 2. A higher subjective value indicates a stronger inclination toward delayed rewards. Then, we collected demographic information such as age and gender. Prior to disclosing the true purpose of the experiment to participants in the final debriefing, we administered a post-experimental question asking whether they had inferred the study’s objective. At the end of the experiment, participants were informed of the true purpose of this study. Additionally, they were informed that they would receive a pen valued at 5 RMB as a token of appreciation.

### 3.3 Results

First, luck manipulation test was conducted. The ANOVA also showed that there were significant differences among the three conditions (The control group served as the good luck control condition) in the luck perception score, *F*(2, 217) = 51.92, *p* < 0.001, *η^2^* = 0.32. Participants in the good luck condition gained luck perception scores (*M* = 5.25, *SD* = 0.91) significantly higher than those obtained in the control condition (*M* = 4.51, *SD* = 1.07; *t*(144) = 4.48, *p* < 0.001, *Cohen’s d* = 0.75) and the bad luck condition (*M* = 3.54, *SD* = 1.11; *t*(148) = 10.38, *p* < 0.001, *Cohen’s d* = 1.70). While participants in the bad luck condition got luck perception score significantly lower than those achieved in the control condition, *t*(142) = −5.37, *p* < 0.001, *Cohen’s d* = 0.89, indicating that the manipulation of luck was effective.

Similarly, the ANOVA also showed that there were significant differences the three conditions (The control group served as the good luck control condition) in the luck perception score, *F*(2, 217) = 49.89, *p* < 0.001, *η^2^* = 0.32. The multiple comparisons showed that participants in the good luck condition gained luck perception scores (*M* = 5.25, *SD* = 0.91) significantly higher than those obtained in the control condition (*M* = 4.59, *SD* = 1.15; *t*(144) = 3.89, *p* < 0.001, *Cohen’s d* = 0.64) and the bad luck condition (*M* = 3.54, *SD* = 1.11; *t*(148) = 10.38, *p* < 0.001, *Cohen’s d* = 1.70). While participants in the bad luck condition got luck perception score significantly lower than those achieved in the control condition, *t*(142) = −5.58, *p* < 0.001, *Cohen’s d* = 0.93, again indicating that the manipulation of luck was effective. There was no significant difference in luck perception between the control groups corresponding to the good luck condition (RMB 75 and RMB 101) and those corresponding to the bad luck condition (RMB 31 and RMB 57), as evidenced by the results (*M* = 4.51, *SD* = 1.07 vs. *M* = 4.59, *SD* = 1.14, *t*(138) = 0.38, *p* > 0.05, *Cohen’s d* = 0.07).

Then, the ANOVA also showed that there were significant differences among the three conditions (The control group served as the good luck control condition) in the results of intertemporal in the decision-making (subjective value of the delay option), *F*(2, 217) = 9.05, *p* < 0.001, *η^2^* = 0.08. The multiple comparisons showed that the subjective value judgment of delay options in the good luck condition (*M* = 71.71, *SD* = 26.90) was significantly higher than that in the control condition (*M* = 57.43, *SD* = 26.62; *t*(144) = 3.22, *p* < 0.001, *Cohen’s d* = 0.53) and the bad luck condition (*M_bad luck_* = 55.54, *SD* = 22.57, *t*(148) = 3.98, *p* < 0.001, *Cohen’s d* = 0.65). There was no significant difference in the subjective value judgment of the delayed option between the participants in the bad luck condition and those in the control condition, *t*(142) = −0.46, *p* > 0.05, *Cohen’s d* = 0.08, indicating that H1 is verified.

Similarly, there were significant differences among the three conditions (The control group served as the bad luck control condition) in the results of intertemporal in the decision-making (subjective value of the delay option), *F*(2, 217) = 8.31, *p* < 0.001, *η^2^* = 0.07. The multiple comparisons showed that the subjective value judgment of delay options in the good luck condition (*M* = 71.71, *SD* = 26.90) was significantly higher than that in the control condition (*M* = 59.43, *SD* = 26.31; *t*(144) = 2.78, *p* < 0.001, *Cohen’s d* = 0.46) and the bad luck condition (*M_bad luck_* = 55.54, *SD* = 22.57, *t*(148) = 3.98, *p* < 0.001, *Cohen’s d* = 0.65). There was no significant difference in the subjective value judgment of the delayed option between the participants in the bad luck condition and those in the control condition, *t*(142) = −0.95, *p* > 0.05, *Cohen’s d* = 0.16, indicating that H1 is verified. In addition, there was no significant difference in intertemporal decision-making behavior between these two sets of control groups (*M* = 59.43, *SD* = 26.41 vs. *M* = 57.43 *SD* = 26.62, *t*(138) = 0.45, *p* > 0.05, *Cohen’s d* = 0.08). Thus, the effect of the numerical anchoring effect on the luck prime can be ruled out and H1 is verified.

## 4 Experiment 2: mediating effects of decision-making confidence

Experiment 2 will improve the external validity of the experiment by changing the luck priming method and the measurement of intertemporal decision making. In addition, Jiang et al. (2009) believe that the priming of luck perception may induce consumers’ positive emotions, such as happiness, optimism. Therefore, the alternative explanation of positive emotions will be excluded in experiment 2.

### 4.1 Purpose and design of the experiment

The purpose of experiment 2 was to test the mediating role of decision-making confidence between luck perception and intertemporal decision-making preferences (H2). A single factor (luck: good luck vs. bad luck) was used as a between-subject experimental design. The sample size calculated by G power was at least 128 (*f* = 0.25, *α* = 0.05, 1 − *β* = 0.80). All participants in this experiment were MBA students with at least 2 years of work experience and had used red envelopes before. The recruitment period for this study was from December 10, 2024, to December 18, 2024. The recruitment method was the same as in experiment 1. In addition, to enhance the validity of the decision-making task, experiment 2 excluded invalid decision choices, such as extreme values that were not in line with common sense when choosing rewards to be received 1 month later (e.g., requesting 500 RMB or more 1 month later, which was regarded as invalid data), as well as participants who did not complete the information. A total of 158 MBA students were recruited from in central China. Of these, 12 participants were excluded due to invalid responses in the decision-making task, resulting in a final sample of 146 valid participants, *M*_age_ = 22.52, *SD* = 2.06; males: 82 (56.20%). Research involving human participants was reviewed and approved by School of Economics and Management, Gandong University. Participants provided written informed consent to participate in this study.

### 4.2 Experimental materials and methods

Firstly, participants were required to accomplish a task of initiating the luck perception. In accordance with the study conducted by Zhao and Zhong (2022), this experiment utilized the method of recalling luck-related events to effectively manipulate the participants’ perception of luck. Specifically, participants were randomly assigned to either the good luck condition or the bad luck condition. Participants in the good luck condition were instructed to recall a fortunate experience or moment (e.g., “obtaining a long-desired blind box”; “winning an extra bottle”), whereas those in the bad luck condition were asked to recollect an unfortunate event or moment (e.g., “a sharp decline in purchased stocks”; “buying counterfeit goods”). All participants were required to provide detailed descriptions in the questionnaire of the specific process of the lucky or unlucky event and their psychological feelings at that time.

Subsequently, participants were informed that they would receive a reward for their participation in the experiment and survey. The reward options were as follows Xu et al. (2019): “To thank you for your support in this survey activity, we will offer you a reward. You can choose to receive it immediately after the session or 1 month later. If you opt for immediate collection, you will receive a 10-yuan (RMB) WeChat red envelope. However, if you prefer to delay receipt by 1 month, please indicate the minimum amount required for you to select this option.” The amount specified by participants reflects the subjective value of the immediate option. The variable “Intertemporal decision-making” in terms of the subjective value of the immediate option. A higher amount indicates a stronger preference for immediate gratification, while a lower amount suggests a greater inclination toward delayed gratification.

After completing the intertemporal decision-making task, participants were required to complete a questionnaire assessing their confidence levels at the moment of making choices (Jiang et al., 2021; Sun et al., 2024; Hock et al., 2020). The decision confidence scale was adapted from Chang et al. (2012) and included items: “I am convinced that I have made the correct choice” “I am certain that my choice represents the optimal one” “I will not be disappointed with my choice” “I have a high level of confidence in my decision—making” “I am highly certain about my decision” (Cronbach’s *α* = 0.85).

Additionally, participants were required to report their positive emotions and luck perception. Positive emotion was measured using items from Jiang et al. (2009), The specific test items are as follows: “I feel optimistic at this moment” “I feel a strong sense of excitement regarding this task” “I take considerable pride in performing this task” “I experience a high level of happiness associated with this task” (1 = strongly disagree, 7 = strongly agree; Cronbach’s *α* = 0.90). Furthermore, participants completed manipulation check items for the luck perception measurement, which were identical to those used in experiment 1 (Cronbach’s *α* = 0.94). Finally, demographic information was collected, and the true purpose of the experiment was disclosed.

### 4.3 Results

Manipulation test. The *t*-test results demonstrated that there were significant differences in the manipulations of luck perception between two conditions. The participants in good luck condition felt luckier (*M_good luck_* = 5.76, *SD* = 1.04) than those in the bad luck condition (*M_bad luck_* = 4.15, *SD* = 1.16), *t*(144) = 8.79, *p* < 0.001, *Cohen’s d* = 1.46. Similarly, decision-making confidence scores differed significantly between the two groups, with higher mean ratings observed in the good luck condition (*M_good luck_* = 5.89, *SD* = 0.93) than in the bad luck condition (*M_bad luck_* = 4.27, *SD* = 0.98), *t*(144) = 10.27, *p* < 0.001, *Cohen’s d* = 1.69. Additionally, intertemporal decision-making results revealed a significant difference in the subjective valuation of immediate rewards between the good luck condition (*M_good luck_* = 43.37, *SD* = 20.34) and the bad luck condition (*M_bad luck_* = 119.24, *SD* = 60.67), *t*(144) = 4.87, *p* < 0.001, *Cohen’s d* = 1.65. Specifically, participants in the good luck condition assigned lower subjective values to immediate rewards, indicating a stronger preference for delayed rewards, which supports H1.

**Figure 2:**
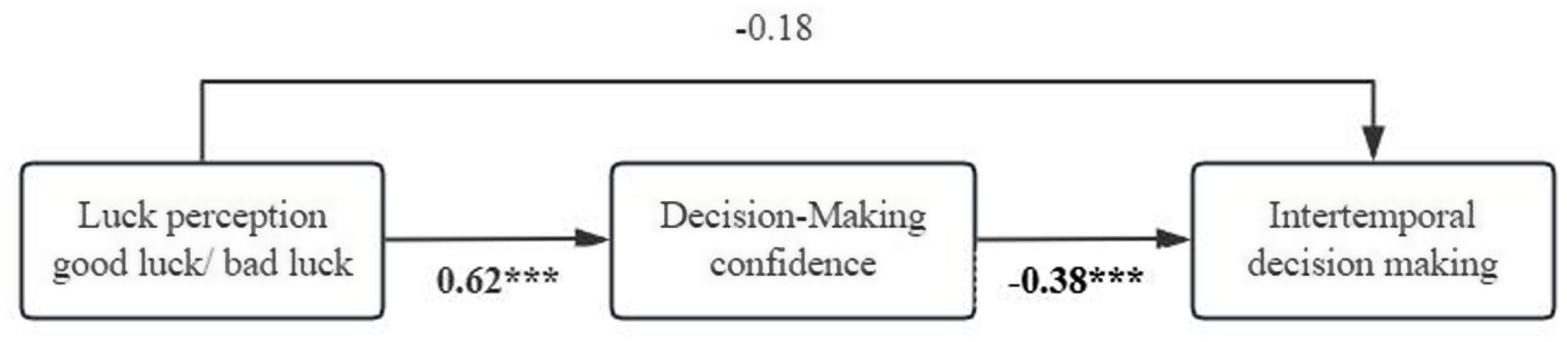
The mediating role of decision-making confidence. ^***^p < 0.001.

Then, variables such as intertemporal decision-making (the subjective value of the immediate option) were standardized. In accordance with the mediation analysis procedure put forward by Hayes (2017), Model 4 of the PROCESS macro (Version 3.5) in SPSS software was adopted to investigate the mediating role of decision-making confidence in the relationship between luck perception and intertemporal decision-making. Luck perception was coded as a dichotomous variable, where 0 represented bad luck and 1 represented good luck. The indirect effect was estimated via 5,000 Bootstrap resamples, and the bias-corrected 95% confidence intervals were computed to evaluate the significance of the mediating effect. The results indicated that the total effect of luck perception on the subjective value of immediate options was −0.41, 95% CI = (−0.5697, −0.2559). The results showed, see Figure 2, that luck perception significantly increased participants’ decision-making confidence [*B* = 0.62, *p* < 0.001, 95% CI = (0.4879, 0.7444)] whereas decision-making confidence decreased the subjective value of immediate options [*B* = −0.38, *p* < 0.001, 95% CI = (−0.5727, −0.1853)]. Decision-making confidence exerted a significant indirect effect in the relationship between luck perception and intertemporal decision-making [*B* = −0.23, *SE* = −0.23, 95% CI = (−0.4699, −0.0449)], but the direct effect of luck perception on intertemporal decision making was not significant, [*B* = −0.18, *p* = 0.06, 95% CI = (−0.3707, 0.0122)], furthermore, the mediating effect of positive emotions was not significant [*B* = −0.01, *p* = 0.71, 95% CI = (−0.0520, 0.0123)]. Therefore, decision confidence fully mediated the effect of luck perception on intertemporal decision making, and H2 was confirmed.

## 5 Experiment 3: the moderating role of self-construal

### 5.1 Purpose and design of the experiment

Experiment 3 was designed to test H3, which posits the moderating effect of self-construal on the relationship between luck perception and intertemporal decision-making preferences. A 2 (luck perception: good luck vs. bad luck) × 2 (self-construal: independent self vs. interdependent self) between-subjects experimental design was utilized. Using G Power, the required sample size was determined to be at least 128 participants (*f* = 0.25, *α* = 0.05, 1 − *β* = 0.80). The participants in experiment 3 were all undergraduate students, and it was required that the subjects had experience in participating in *Douyin* live-streaming activities. The recruitment approach was consistent with that of experiment 1. The recruitment period for this study extended from December 19, 2024, to December 30, 2024. To enhance the validity of both the participants and the decision-making task, experiment 3 excluded invalid decision-making choices. These included cases where participants withdrew from the experiment midway, data of participants with incomplete information, and situations where the filled-in amount for the delayed reward was unrealistic, presenting extreme values that deviated from common sense (e.g., 1,000 MB). A total of 138 undergraduate students from a university in central China were recruited. Among them, 8 students were excluded because their choices in the decision-making task were invalid. As a result, the number of valid participants was 130, *M_age_* = 20.41, *SD* = 1.05, males: 66 (50.77%). Research involving human participants was reviewed and approved by School of Economics and Management, Gandong University. Participants provided written informed consent to participate in this study.

### 5.2 Experimental materials and methods

Before the experiment began, participants were informed that they would complete three ostensibly unrelated experimental tasks. The first task involved a situational assignment designed to prime self-construal and luck perception; the second was a consumption choice experiment; and the third was a psychological questionnaire.

Existing studies often manipulate long-term self-construal by using culturally distinct samples from Eastern and Western contexts (e.g., Chinese samples for independent self-construal and American samples for interdependent self-construal). However, due to practical constraints, all participants in this study were recruited from a Chinese population (Duan et al., 2018). Despite this limitation, prior research has demonstrated that self-construal primed through situational stimuli can exert effects comparable to those of long-term self-construal (Trafimow et al., 1991). Therefore, this experiment employed a situational approach to prime participants’ self-construal levels. During the experiment, participants were randomly assigned to one of two conditions: an independent self-construal priming condition or an interdependent self-construal priming condition.

First, in the independent individual group, participants were presented with the following materials: “The more one admires it, the higher it appears; the deeper one explores it, the harder it becomes.” The pursuit is not only to become a champion but also to transcend the title of champion. Once an era begins, its conclusion is rarely straightforward or effortless. Although the achievements of predecessors are remarkable and the potential of newcomers is undeniable, one must never underestimate the determination of a champion. As long as you possess the will to compete, every moment can be your peak time. Drawing from the words of Confucius, “Even the great sage may be in awe of the younger generation, and no man should look down upon the young.” Similarly, as expressed in the oath of a certain middle school, “I was born to be a mountain, not a stream; I aspire to overlook the mundane valleys from the summit of towering peaks. I was born to be an extraordinary person, not insignificant grass. Standing on the shoulders of giants, I disdain mediocrity and weakness!” When your capabilities are fully developed, you can soar to the highest peak, claim the olive branch, and crown yourself. Participants were tasked with identifying pronouns such as “he,” “you,” “I,” and other terms symbolizing or describing individuals.

In the dependent individual group, participants were provided with the following textual materials: Chinese traditional culture emphasizes social harmony and interpersonal coexistence. An individual cannot exist independently of others, as interpersonal relationships play a crucial role in shaping personal identity. This cultural framework underscores shared experiences, emotional connections, and the fulfillment of social responsibilities. At the Paris Olympics, the Chinese men’s swimming team secured the gold medal in the 4 × 100-m medley relay through the collective efforts of all team members. Following the event, Pan Zhanle, the renowned “flying fish” in the 100-m race, remarked, “While an individual can swim fast alone, a team can achieve even greater speeds together! I am not fighting alone; behind me stands a powerful nation.” In a similar vein, during the 41st year since the establishment of the Chinese synchronized swimming team, athletes dedicated nearly 15,000 days and nights to transforming collective dreams into reality. Team captain Feng Yu stated, “This gold medal belongs not only to our team but also to generations of Chinese synchronized swimmers who have paved the way. It carries profound significance.” Unity, inheritance, responsibility, and perseverance are the cornerstones of the Chinese delegation’s remarkable achievements. Participants were tasked with identifying pronouns were instructed to identify words such as “we,” “they,” and other terms symbolizing collectivity. Subsequently, participants in the independent self-construal condition were asked to carefully reflect and write down three personal expectations, while participants in the interdependent self-construal condition were asked to write down three expectations from family members or friends. To verify the effectiveness of the self-construal priming, participants completed self-construal measurement scale (Singelis, 1994) after completing the above tasks. Nine items for interdependent self-construal, Cronbach’s *α* = 0.89, the specific scales are as follows: “In interpersonal interactions, individuals should be attentive to preserving others’ face.” “Upon joining a group, individuals are expected to actively conform to its requirements.” “I often find that maintaining harmonious interpersonal relationships is more important than achieving personal success.” “Respecting collective decisions is highly important to me.” “Even when experiencing negative emotions, I will remain with the group if it requires my presence.” “Even in cases of strong disagreement with the majority opinion within a team, I tend to avoid confrontation.” “Maintaining harmonious interpersonal relationships is of fundamental importance to me.” “When making educational or career decisions, I consider my parents’ recommendations.” “I am willing to sacrifice personal interests for the benefit of the collective.” 9 items for independent self-construal, Cronbach’s *α* = 0.85, the specific scales are as follows: “Rather than being misunderstood, I prefer to express my thoughts directly and explicitly.” “I experience comfort when I receive individual praise or rewards.” “Speaking in class poses no significant challenge for me.” “Maintaining an active imagination is of great significance to me.” “Taking good care of myself constitutes my primary concern.” “My performance remains consistent regardless of the company I keep.” “When interacting with newly acquainted individuals, I tend to be direct and forthright.” “I prefer to distinguish myself in multiple aspects.” “Having an identity independent of others is of crucial importance to me.”

Next, participants’ luck perception was primed through a simulated lottery game (Zhao, 2023). Each participant had one chance to draw a prize using custom-designed wheel-of-fortune software. For the luck group, participants were told that the winning probability was 0.1 (in reality, the probability was set to 1, ensuring all participants won), and upon winning, they saw a message displayed for 5 s: “Congratulations! You are very lucky and have won!” For the bad-luck group, participants were told that the winning probability was 0.6 (in reality, the probability was set to 0, ensuring no participants won), and they saw a message displayed for 5 s: “Unfortunately, you did not win.”

Following the lottery task, participants completed a “consumption choice” survey. This survey consisted of two parts. One part assessed participants’ intertemporal decision-making behavior, focusing on their preferences for immediate versus delayed rewards. Participants were presented with a hypothetical scenario: Imagine you have won a 100-yuan (RMB) cash coupon in a *Douyin* live stream that can be redeemed immediately for cash. However, due to logistical delays, the live stream offers an alternative option to redeem a higher-value coupon after 6 months. Participants were asked to indicate the minimum amount of the delayed coupon they would accept. The measurement of intertemporal decision-making was consistent with experiment 2.

The final part of the survey assessed participants’ decision confidence (Cronbach’s *α* = 0.85), luck perception, and positive emotions (Cronbach’s *α* = 0.90). Positive emotions were measured using the same scale as in experiment 2. Luck perception was assessed using a single-item manipulation check: “I feel I have good luck today” (rated on a 7-point Likert scale, where 1 = strongly disagree and 7 = strongly agree). Finally, demographic information was collected. After completing the experiment, participants were debriefed regarding its true purpose and thanked with a pen as a token of appreciation.

### 5.3 Results

Luck manipulation Test. t-test results showed that there was a significant difference between participants in the good luck condition and the bad luck condition in terms of luck perception (*M_good luck_* = 5.53, *SD* = 0.83, *M_bad luck_* = 3.94, *SD* = 0.77, *t*(128) = 11.34, *p* < 0.001, *Cohen’s d* = 1.98). In addition, there was no significant difference between participants in the good luck condition and the bad luck condition in terms of positive emotions (*M_good luck_* = 5.45, *SD* = 1.23, *M_bad luck_* = 5.21, *SD* = 1.22, *t*(128) = 1.17, *p>* 0.05, *Cohen’s d* = 0.19).

Individuals with an independent self-construal demonstrated a significantly greater perception of independence compared to those with an interdependent self-construal, as evidenced by the experimental findings, (*M*_*independent self-construa*l_ = 5.76, *SD* = 0.57, VS. *M_interdependent self-construal_* = 4.20, *SD* = 0.67, *t*(128) = 14.10, *p* < 0.001, *Cohen’s d* = 2.49). At the same time, individuals with an dependent self-construal reported a significantly stronger sense of dependence compared to those with an independent self-construal, as indicated by the experimental results, (*M_interdependent self-construal_* = 5.80, *SD* = 0.46, VS. *M*_*independent self-construa*l_ = 4.72, *SD* = 0.73, *t*(128) = 10.14, *p* < 0.001, *Cohen’s d* = 1.78).

**Figure 3:**
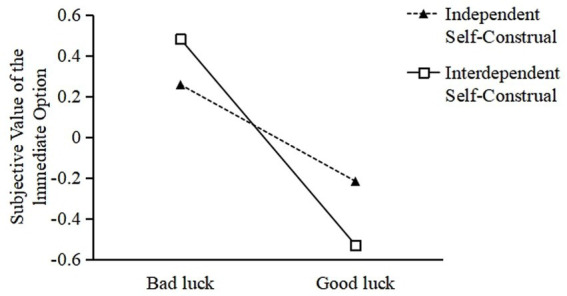
Influence of luck perception and self-construal on subjective value of the immediate option.

Luck perception was set as the independent variable, decision confidence as the mediating variable, subjective value of the immediate option (with higher monetary amounts indicating a stronger preference for immediate rewards and lower amounts reflecting a greater inclination toward delayed rewards) as the dependent variable, and self-construal as the moderating variable. Furthermore, the variables such as the subjective values of the immediate options have been standardized. In accordance with the mediation analysis procedure put forward by Hayes (2017), the Process 3.5 macro in SPSS was used, with Model 8 selected, and a Bootstrap procedure conducted using 5,000 samples at a 95% confidence interval. Firstly, H3 was tested and validated. The results demonstrated that the interaction between luck perception and self-construal significantly affected intertemporal decision-making, [*B* = −0.54, *SE* = 0.26, *p* < 0.05, 95% CI = (−1.0501, −0.0217)]. In this paper, we conduct a simple slope analysis following the research of Preacher and Hayes (2008). The findings reveal that among individuals with an interdependent self-construal, the direct effect of luck perception on intertemporal decision-making is statistically significant [*B* = −1.0, *SE* = 0.25, *p* < 0.001, 95% CI = (−1.5103, −0.5085)], and for individuals with an independent self-construal, the direct effect of luck perception on intertemporal decision-making is significantly reduced, [*B* = −0.47, *SE* = 0.23, *p* = 0.04, 95% CI = (−0.9319, −0.0151)]. The simple slope analysis chart (see Figure 3) more explicitly demonstrates that, in contrast to individuals with an independent self-construal, the effect of luck perception on intertemporal decision-making is more salient among individuals with an interdependent self-construal, thereby validating H3.

**Figure 4:**
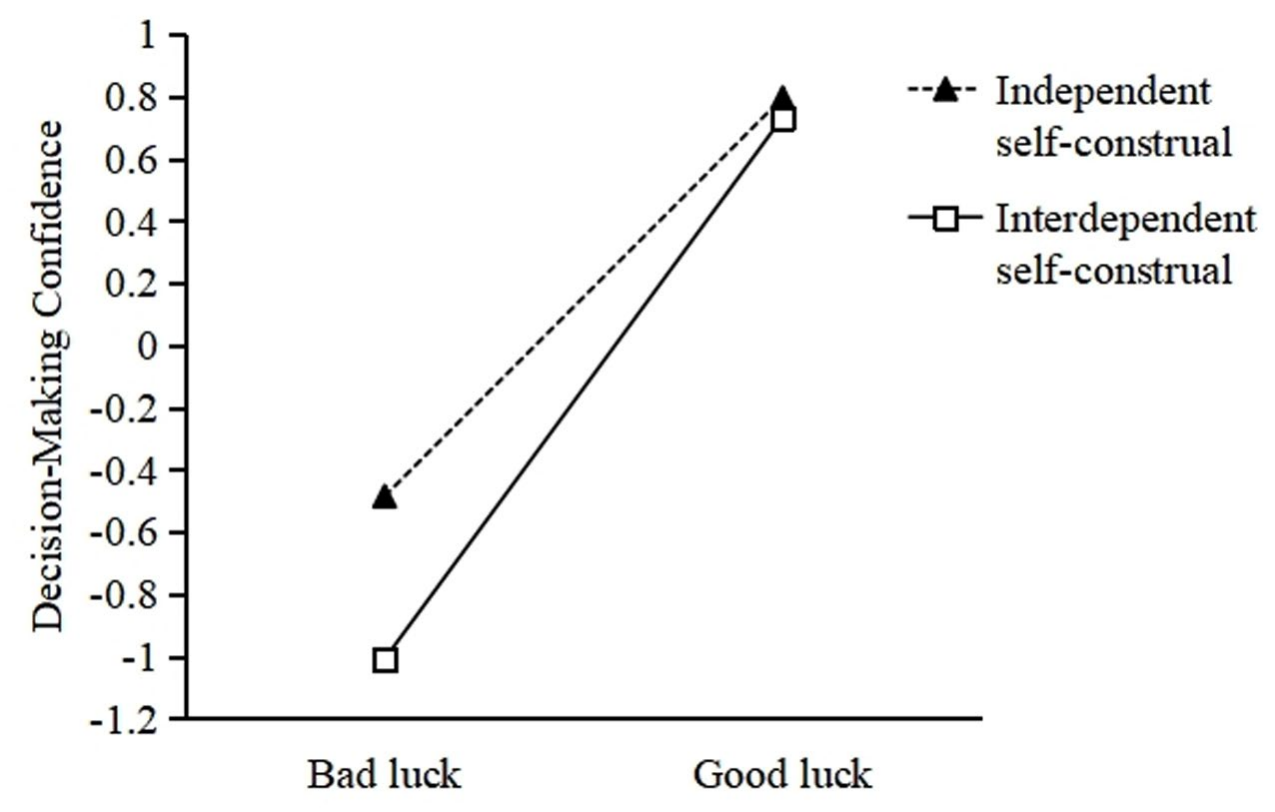
Influence of perceived luck and self-construal on decision-making confidence.

Second, the results also found that the interaction term between perceived luck and self-construal had a significant effect on decision-making confidence [*B* = 0.46, *SE* = 0.21, *p* = 0.03, 95% CI = (0.0360, 0.8842)]. The simple slope analysis (Figure 4) indicates that among individuals with an interdependent self-construal, the positive association between luck perception and decision confidence is statistically significant, [*B* = 1.74, *SE* = 0.15, *p* < 0.001, 95% CI = (1.4534, 2.0286)]. Among individuals with independent self-construal, the positive impact of luck perception on decision—making confidence would be substantially attenuated [*B* = 1.28, *SE* = 0.16, *p* < 0.001, 95% CI = (0.9693, 1.5926)].

Finally, a moderated mediation analysis was conducted. As indicated in Table 1, for individuals with interdependent self-construal, luck perception strengthens decision-making confidence, thereby increasing the tendency to choose delayed options (reducing the subjective value of the immediate option). For individuals with independent self-construal, the effect of luck perception on reducing the subjective value of the immediate option via the mediating role of decision confidence is diminished. Additionally, the difference in this moderated mediation effect is statistically significant. Therefore, H4 is supported.

**Table 1:** Test of moderated mediation effect.

Self-construal	Luck → confidence in decision making → subjective value of immediate option		
	Indirect effect	Boot LLCI	Boot ULCI
Independent self-construal	−0.48^***^	−0.6791	−0.2789
Interdependent self-construal	−0.65^***^	−0.9096	−0.3734
Difference value	−0.17^*^	−0.3848	−0.0087

^***^p < 0.001, ^**^p < 0.01, ^*^p < 0.05.

## 6 Discussion

This paper investigates the influence of luck perception on individuals’ decision-making behaviors in intertemporal decision-making under uncertainty, focusing on immediate options (smaller-sooner) versus delayed options (larger-later), the underlying psychological mechanisms, and moderating conditions. First, compared to the bad luck condition and control condition, the good luck condition exhibits a stronger preference for larger delayed options in intertemporal decision-making tasks. Second, decision confidence mediates the effect of luck perception on intertemporal decision-making. Specifically, the good luck condition demonstrates higher decision confidence than the bad luck group, leading to a greater preference for delayed gratification. Third, the moderating role of self-construal is confirmed. Luck perception has a more pronounced impact on decision confidence and intertemporal decision-making among individuals with an interdependent self-construal compared to those with an independent self-construal. Additionally, self-construal directly moderates the effect of luck perception on intertemporal decision-making. Prior studies have primarily examined the control perspective, suggesting that luck perception helps individuals fulfill their need for control, reduce stress and anxiety, and facilitate rapid decision-making in uncertain or risky situations (Lim and Rogers, 2017; Lasikiewicz and Teo, 2018). However, these studies overlooked other needs, such as the desire for high-value or high-benefit outcomes. Tao et al. (2023) found that individuals who feel lucky exhibit high-level explanatory characteristics and promotion orientation, focusing on overall value and long-term benefits while paying less attention to decision-making details. Building on this foundation, this study reveals through experimental research that decision-makers perceiving themselves as lucky are more patient in intertemporal tasks and tend to select large and distant delayed options. This conclusion aligns with prior findings that associate luck with high control needs and high-level explanations, integrating uncertainty risk and promotion orientation perspectives and extending luck research into intertemporal decision-making under uncertainty.

### 6.1 Theoretical significance

This study contributes to the expanding literature on intertemporal decision-making and luck perception. While traditional theories of intertemporal decision-making have largely emphasized rational models such as time discounting, the present research introduces “luck perception” as a subjective cognitive factor and examines its potential role in shaping delayed gratification through enhanced decision confidence. This finding offers a novel, albeit preliminary, theoretical perspective on the influence of non-rational factors in intertemporal choices, suggesting that perceived luck may function as a cognitive resource that helps individuals manage uncertainty in decision-making contexts.

Second, this study provides initial empirical support for the moderating role of self-construal. Results indicate that the positive effect of luck perception on decision confidence and intertemporal decision-making is significantly stronger among individuals with an interdependent self-construal. This aligns with and extends established theory—namely, that interdependent selves are more responsive to contextual cues—by demonstrating its applicability within the domain of luck-related cognition. Specifically, the findings suggest that individuals with an interdependent orientation may be more skilled at transforming external cues of fortune into optimistic future expectations. As such, this research advances understanding of the boundary conditions under which self-construal exerts influence on decision processes.

Finally, this work extends the investigation of luck perception from risk-based decisions into the domain of intertemporal decision-making. In contrast to prior research focusing on luck’s effects on risk-taking or control restoration, the current study explores how luck perception operates in a context involving temporal delay rather than outcome uncertainty. By identifying decision confidence as a plausible mediating mechanism, this research not only highlights a new pathway linking luck to choice behavior but also lays the groundwork for future studies aiming to uncover the broader psychological mechanisms through which luck shapes decision-making across different domains.

### 6.2 Practical implications

Firstly, this study offers practical insights into how individuals make decisions under uncertainty and the role of luck perception in shaping these decisions. It highlights the importance of considering subjective factors like luck perception in decision-making processes, particularly in contexts involving long-term planning and delayed gratification. The findings can inform strategies to enhance decision-making confidence and improve long-term decision-making capabilities.

Moreover, the emphasis on the moderating role of self-construal enables tailored decision-making interventions for individuals with different self-construal orientations, potentially improving personalized decision-making support. Overall, this research contributes to understanding the complex interplay between subjective perceptions and decision-making behaviors, with applications in finance, marketing, and personal development. For example, in education, interventions can be designed to enhance students’ delayed gratification abilities by combining positive psychology courses (e.g., “lucky diaries”) with interdependent self-construal characteristics (e.g., team collaboration tasks). In financial decision-making, personalized guidance can be provided based on consumers’ luck perception tendencies and self-construal types. For interdependent individuals, emphasizing the connection between “lucky opportunities” and long-term returns may enhance their patience in holding high-potential assets. For independent individuals, focusing on rational risk analysis can bridge the confidence gap.

Finally, to promote mental health and address immediate gratification behaviors (e.g., game addiction or hedonic consumption), the “luck perception—decision confidence” pathway can be leveraged. Luck priming methods can assist individuals in reconstructing future preferences and resisting immediate temptations. For interdependent individuals, family or community support systems can amplify intervention effects.

### 6.3 Limitations

This study has several limitations that point to valuable directions for future research. In terms of methodology, the present research has a few notable limitations. First, regarding cultural generalizability, the luck priming cues based on the numbers “8” and “4” used in experiment 1 are deeply rooted in Chinese cultural and phonetic-semantic associations, which may restrict the cross-cultural applicability of the findings. To improve external validity, future studies could adopt cross-cultural experimental designs or employ non-linguistic symbolic cues—such as lucky charms or visual icons—that are less dependent on specific language contexts. Second, in experiment 1, the priming task (power bank pricing) and the dependent measure (Weibo lottery) were administered in separate contexts. Although this design was intended to test the generalizability of effects across domains, it may have introduced demand characteristics, whereby participants inferred the research purpose and adjusted their responses accordingly. To address this, we excluded the small number of participants who accurately guessed the hypothesis during data analysis. Future research would benefit from adopting more implicit measurement techniques or conducting field experiments to minimize such biases and enhance the robustness of the findings. Furthermore, in experiment 3, a narrative-based priming method was used to enhance ecological validity and intensify the activation of self-construal, resulting in a relatively complex procedure. Although this approach helped elicit more natural and sustained shifts in self-representation, it may have increased participants’ cognitive load and procedural complexity, raising concerns about participant burden and potential confounding influences. To strengthen methodological rigor, future studies could employ well-validated and concise priming tasks—such as the pronoun circling task—to replicate the current findings. Such methods offer better experimental control, reduce the unintended activation of extraneous constructs, and help facilitate cross-validation across simpler paradigms, thereby improving both internal validity and methodological reliability.

Regarding decision-making contexts and theoretical mechanisms, several gaps remain open for further exploration. First, the current research focused exclusively on intertemporal choices in gain frames, leaving unexamined the role of luck perception in loss contexts. Future research should investigate how luck perception influences decision-making and underlying psychological processes when individuals face potential losses. Second, a growing body of research has established self-control as a key psychological mechanism underlying intertemporal decision-making (He and Yan, 2015). At the same time, luck perceptions have been shown to influence individuals’ sense of control and the availability of associated regulatory resources. Furthermore, uncertainty tolerance is recognized as a salient individual difference factor that shapes intertemporal choices (Epper et al., 2011; Boelen et al., 2014). Individuals who perceive themselves as unlucky often report diminished confidence and a heightened sense of control deprivation, which are associated with lower tolerance for uncertainty and a tendency to overestimate both the likelihood and severity of negative outcomes. In contrast, those who perceive themselves as lucky tend to believe that favorable outcomes are within their reach and interpret luck as an extension of personal agency. This perception enables them to transform luck into a potent psychological resource that enhances perceived control, thereby increasing their willingness to wait for delayed rewards. Given these interrelated constructs, future research should systematically examine the interplay among uncertainty tolerance, luck perception, self-control, and intertemporal decision-making to clarify the underlying mechanisms.

## Data Availability

The original contributions presented in the study are included in the article/supplementary material, further inquiries can be directed to the corresponding author.
